# High-yield isolation of menstrual blood-derived endometrial stem cells by direct red blood cell lysis treatment

**DOI:** 10.1242/bio.038885

**Published:** 2019-04-29

**Authors:** Yuliang Sun, Yakun Ren, Fen Yang, Yanan He, Shengying Liang, Lihong Guan, Fangfang Cheng, Yanli Liu, Juntang Lin

**Affiliations:** 1 Stem Cell Research Center, College of Life Science and Technology, Xinxiang Medical University, Xinxiang 453003, China; 2 Henan Key Laboratory of Medical Tissue Regeneration, Xinxiang 453003, China; 3 College of Biomedical Engineering, Xinxiang Medical University, Xinxiang 453003, China

**Keywords:** Adult stem cells, Menstrual blood-derived endometrial stem cells, Stem cell based therapy, High-yield, Red blood cell lysis

## Abstract

Recently, menstrual blood-derived endometrial stem cells (MenSCs) have become attractive for stem cell based therapy due to their abundance, easy and non-invasive extraction and isolation process, high proliferative capacity, and multi-lineage differentiation potential. MenSC-based therapies for various diseases are being extensively researched. However, the high death rate and poor engraftment in sites of damaged tissues reduce the therapeutic value of these stem cells for transplantation. In theory, periodic stem cell transplantation is an alternative strategy to overcome the challenge of the loss of beneficial stem cell-derived effects due to the rapid disappearance of the stem cells *in vivo*. However, periodic stem cell transplantation requires sufficient amounts of the desired stem cells with a low number of subculture passages. Our previous results have demonstrated that primary MenSCs mainly reside in the deciduous endometrium, and considerable amounts of deciduous endometrium intertwined with menstrual blood clots were discarded after conventional density gradient centrifugation (DGC). Therefore, the aim of this study was to determine whether primary MenSCs exist in the sedimentation of the deciduous endometrium after DGC and further to evaluate the isolation of MenSCs by direct red blood cell lysis treatment. As expected, our results confirmed that substantial amounts of primary MenSCs still remain in the sedimentation after DGC and indicated that MenSC isolation by directly lysing the red blood cells not only guaranteed substantial amounts of superior MenSCs with a low number of subculture passages, but also was time efficient and economical, providing a solid support for extensive clinical application.

## Abstract (precis)

**Summary:** MenSC isolation by directly lysing the red blood cells not only guarantees substantial amounts of superior MenSCs with low passage number, but also is time efficient and economical.

## INTRODUCTION

Over the past decade, stem cell based therapies have gradually advanced from preclinical studies to clinical validation studies, and the results of numerous clinical trials look encouraging, especially those from adult stem cell (ASC)-based therapies (Mirzaei et al., 2018; Vogel, 2014; Segers and Lee, 2008). However, tracking stem cells *in vivo* has indicated that only a minor proportion of cells reside and persist in the target sites due to the unfavorable conditions of damaged tissue, and the majority of stem cells are not detectable 2 weeks after transplantation, significantly reducing the efficacy of stem cell based therapy (Kurtz, 2008; Liu et al., 2018b). It seems that the therapeutic effects of ASC transplantation were mainly realized via the paracrine effect and immunomodulation within short periods of time, rather than transdifferentiation or engraftment to target sites in significant numbers (Jiang et al., 2013; Ranganath et al., 2012; Chen et al., 2017). Therefore, a sufficient quantity of ASCs with low numbers of subculture passages is indispensable for guaranteeing the therapeutic effect of ASC transplantation.

Menstrual blood-derived endometrial stem cells (MenSCs), as newly identified ASCs, have been demonstrated to reside in the basal layer of the endometrium and can be isolated from menstrual blood. Since their discovery in 2007, MenSCs have exhibited outstanding advantages such as abundance, easy and non-invasive extraction and isolation process, high proliferative capacity and multi-lineage differentiation potential (Meng et al., 2007; Liu et al., 2018a; Gargett et al., 2015). Previous studies have confirmed that MenSCs positively expressed the surface markers of mesenchymal stem cells, such as CD29, CD44, CD73, CD90 and CD105 and are negative for hematopoietic cell markers, including CD34 and CD45 (Gargett et al., 2015; Patel et al., 2008). Furthermore, several pluripotent stem cell markers, such as OCT-4, c-kit and SSEA-4, are also positively expressed by MenSCs (Liu et al., 2018a; Masuda et al., 2010). Currently, MenSCs can be successfully differentiated into various cell lineages derived from all three germ layers (ectoderm, endoderm and mesoderm), including neurogenic cell, cardiac cell, osteoblasts, chondrocytes, adipocytes and hepatocytes (Gargett et al., 2015; Ulrich et al., 2013; Liu et al., 2018a,c). More importantly, in addition to promising therapeutic effects in animal disease models, clinical trials have also confirmed the therapeutic effects and safety of MenSC transplantation (Ichim et al., 2010; Bockeria et al., 2013).

The current conventional MenSC isolation process is based on density gradient centrifugation (DGC), which unfortunately leaves clearly observable karyocytes and flocculent membranes (deciduous endometrium) in the buffy coat, yet generally, it is the deciduous endometrium that produces the most MenSC clones (Meng et al., 2007; Patel et al., 2008). After the standard DGC procedure, we found that the deciduous endometrium is often intertwined with menstrual blood clots that are separated by centrifugation and discarded as waste. We reasonably supposed that this outcome likely causes a considerable loss in the portion of primary MenSCs that could be collected during conventional DGC isolation. Therefore, the aim of this study was to confirm whether, after DGC, there are primary MenSCs remaining in the sedimentation in which the deciduous endometrium is intertwined with menstrual blood clots. We further aimed to evaluate the production of MenSCs by direct red blood cell lysis treatment. The optimization of MenSCs isolation not only maximizes the value of menstrual blood samples but also provides a substantial number of superior MenSCs (with low number of subculture passages) for extensive application in clinical trials.

## RESULTS

### Isolation and identification of MenSCs

**Fig. 1.**
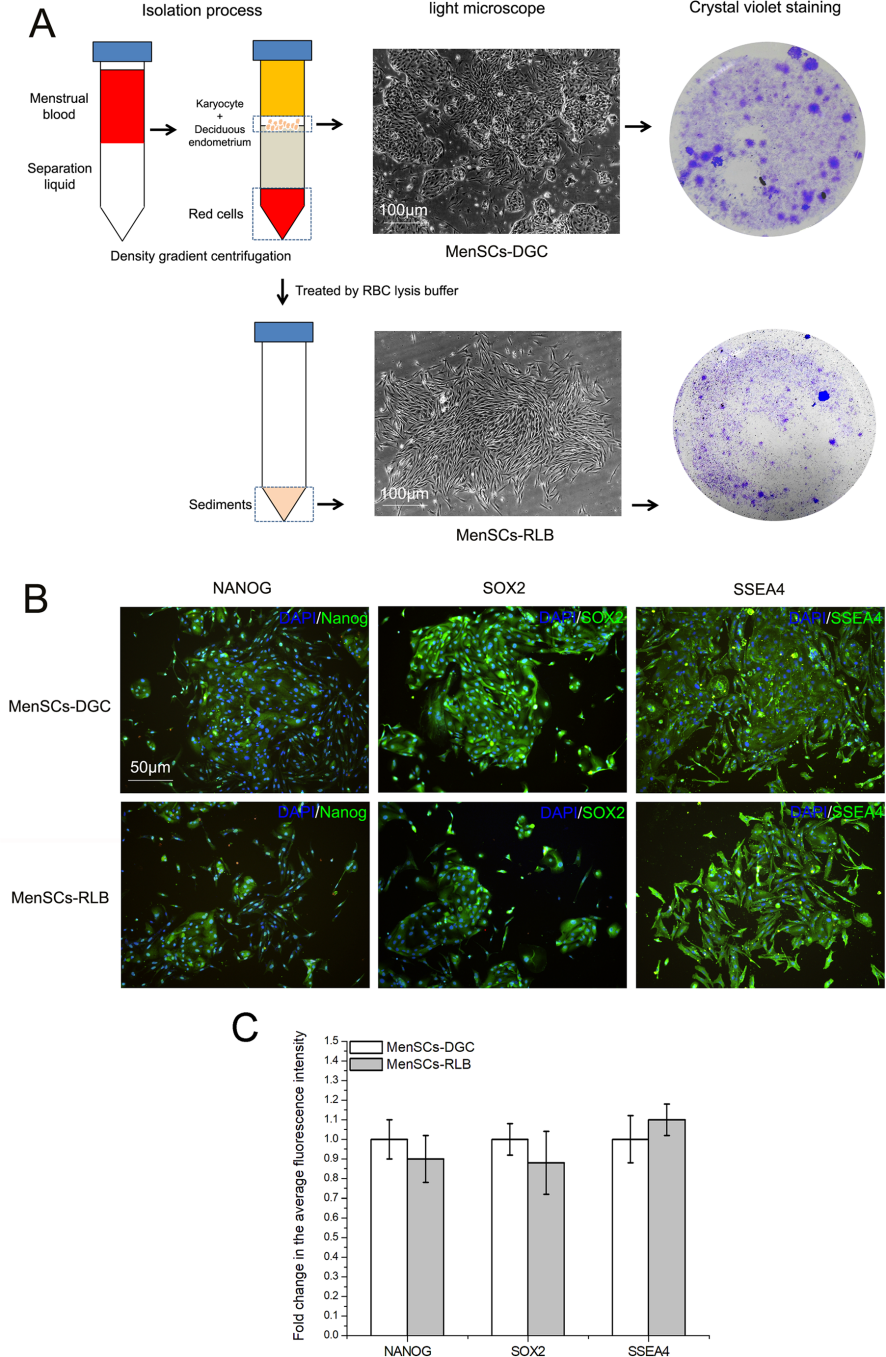
**Considerable amount of primary MenSCs remain in the sedimentation after conventional DGC.** (A) The isolation procedures and morphology of primary MenSCs-DGC and MenSCs-RLB. After conventional DGC, the karyocytes and deciduous endometrium suspended in the buffy coat layer were collected, washed, resuspended and cultured in medium. Simultaneously, the sedimentation after DGC was treated with red blood cell (RBC) lysis buffer twice, and then the remaining sedimentation was washed, resuspended and cultured in medium. After 5 days of culture, the morphology of MenSC-formed clones was imaged under an inverted microscope and macroscopically observed after Crystal Violet staining. (B) Both MenSCs-DGC (*n*=3) and MenSCs-RLB (*n*=3) express typical ESC markers. After 5 days of culture, primary MenSCs were subjected to immunofluorescent staining of Nanog, SOX2 and SSEA4. (C) Quantification of the expression of Nanog, SOX2 and SSEA4 in MenSCs-DGC and MenSCs-RLB. Scale bars: 100 μm in A; 50 μm in B.

**Fig. 2.**
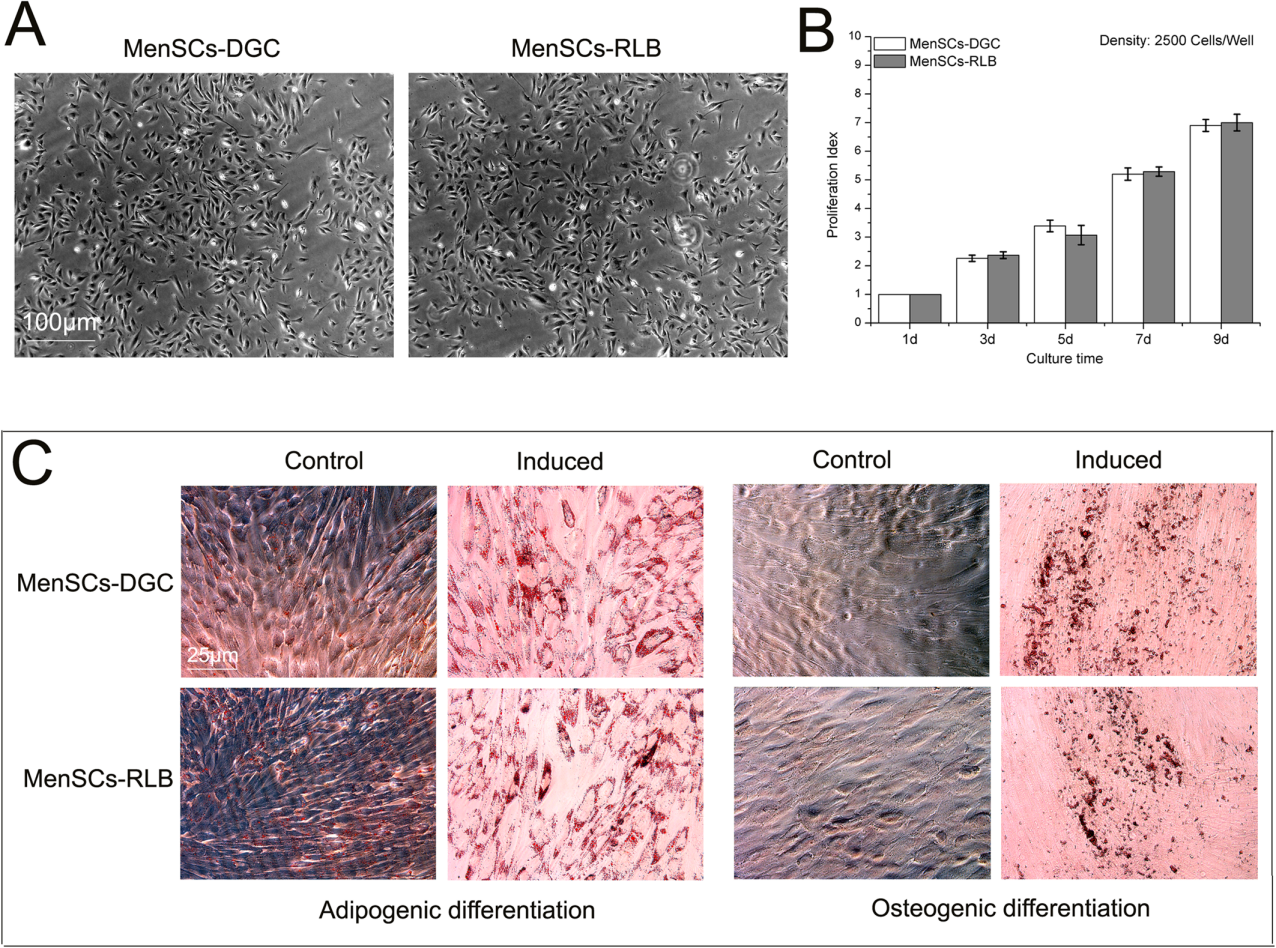
**The morphology, proliferation capacity and multidifferentiation potential of MenSCs.** (A) The representative morphology of P3 MenSCs-DGC (*n*=3) and MenSCs-RLB (*n*=3) were imaged under an inverted microscope. (B) P3 MenSCs-DGC and MenSCs-RLB were seeded into 96-well plates with indicated cell density, and the proliferation capacity during the 9-day culture was determined by MTT assay. The PI are presented as fold-change relative to the absorbance of MenSCs cultured for 1 day. (C) P3 MenSCs-DGC and MenSCs-RLB were used to perform conventional adipogenic and osteogenic differentiation and the results were visualized by positive Oil Red O and Alizarin Red staining, respectively. Scale bars: 100 μm in A; 25 μm in C.

**Fig. 3.**
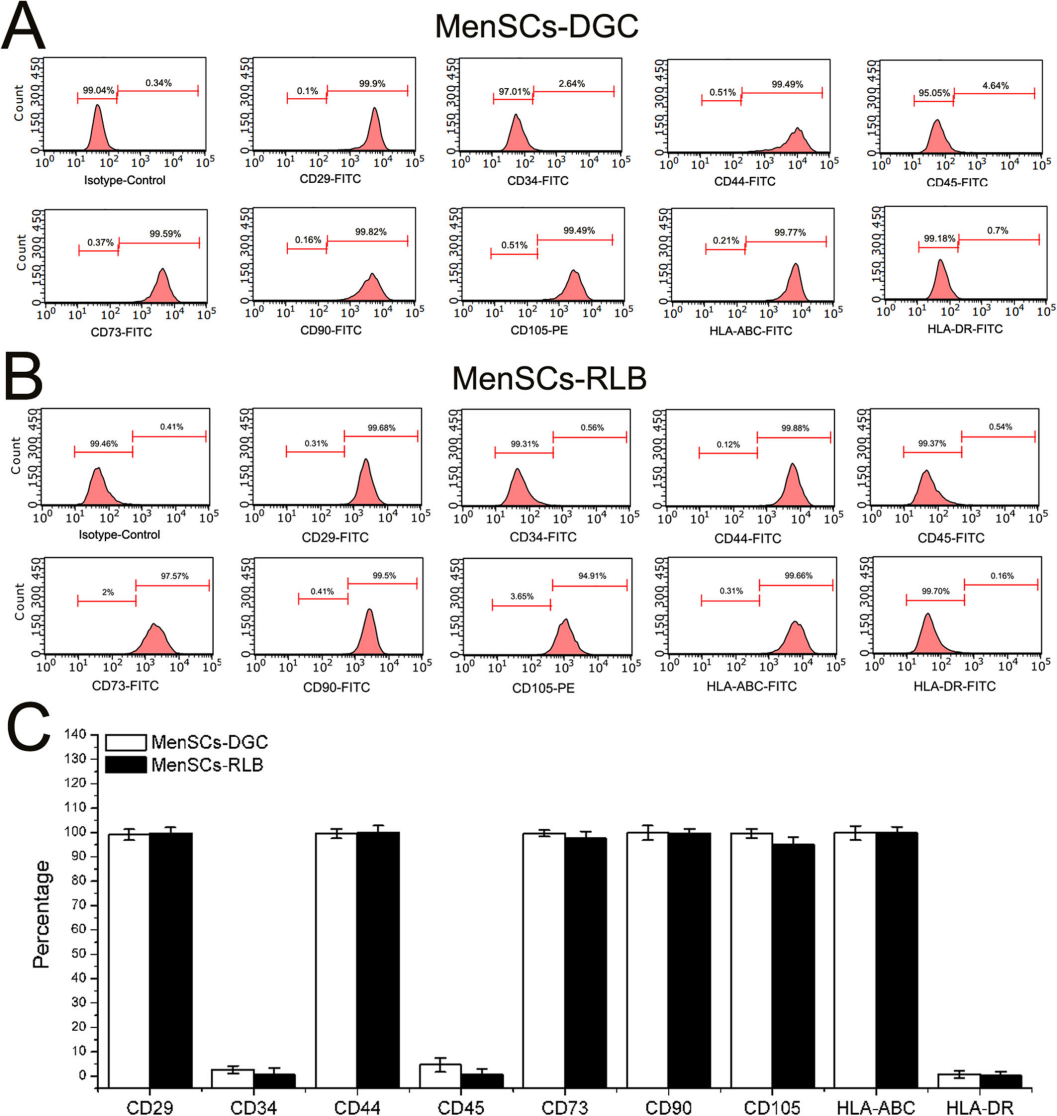
**The phenotype of MenSCs.** (A,B) To determine the immunophenotype of MenSCs, P3 MenSCs-DGC (*n*=5) and MenSCs-RLB (*n*=5) were stained by corresponding conjugated antibodies and analyzed by FACS. Both MenSCs-DGC and MenSCs-RLB positively expressed classical ASCs' markers (CD29, CD44, CD73, CD90 and CD105) and HLA-ABC; they did not express hematopoietic stem cell markers (CD34 and CD45) and HLA-DR. (C) The quantification of flow cytometry results of A and B.

During the primary culture period (Passage 0, P0), both MenSCs formed colonies with radial or helix growth patterns (Fig. 1A and below), and after subculturing, the MenSCs displayed a typical spindle fibroblast-like morphology during the whole *in vitro* culture period (Fig. 2A). Subsequently, our results demonstrate that all the MenSCs isolated in the study fulfill the standards for adult stem cells. Both MenSCs-DGC and MenSCs-RLB were positive for CD29, CD44, CD73, CD90, CD105 and HLA-ABC, but were negative for CD34, CD45 and HLA-DR, and no significant difference was observed between either (Fig. 3). Simultaneously, multi-lineage differentiation assays also confirmed that both MenSCs could undergo adipogenic and osteogenic differentiation after being treated with specific induction media (Fig. 2C). As shown in Fig. 1B,C, both MenSCs-DGC and MenSCs-RLB positively expressed Nanog, SOX2 and SSEA4, which are typical embryonic stem cell markers, suggesting superior multipotency, and their expression levels had no statistical difference between MenSCs-DGC and MenSCs-RLB.

### Proliferative capacity

The cell viability of P3 MenSCs was examined by a conventional MTT assay. As shown in Fig. 2B, robust growth trends were observed in both the MenSCs-DGC and MenSCs-RLB as the culture time increased. No significant difference was observed in the cell viability between MenSCs-DGC and MenSCs-RLB.

### MenSCs isolation efficiency analysis

**Fig. 4.**
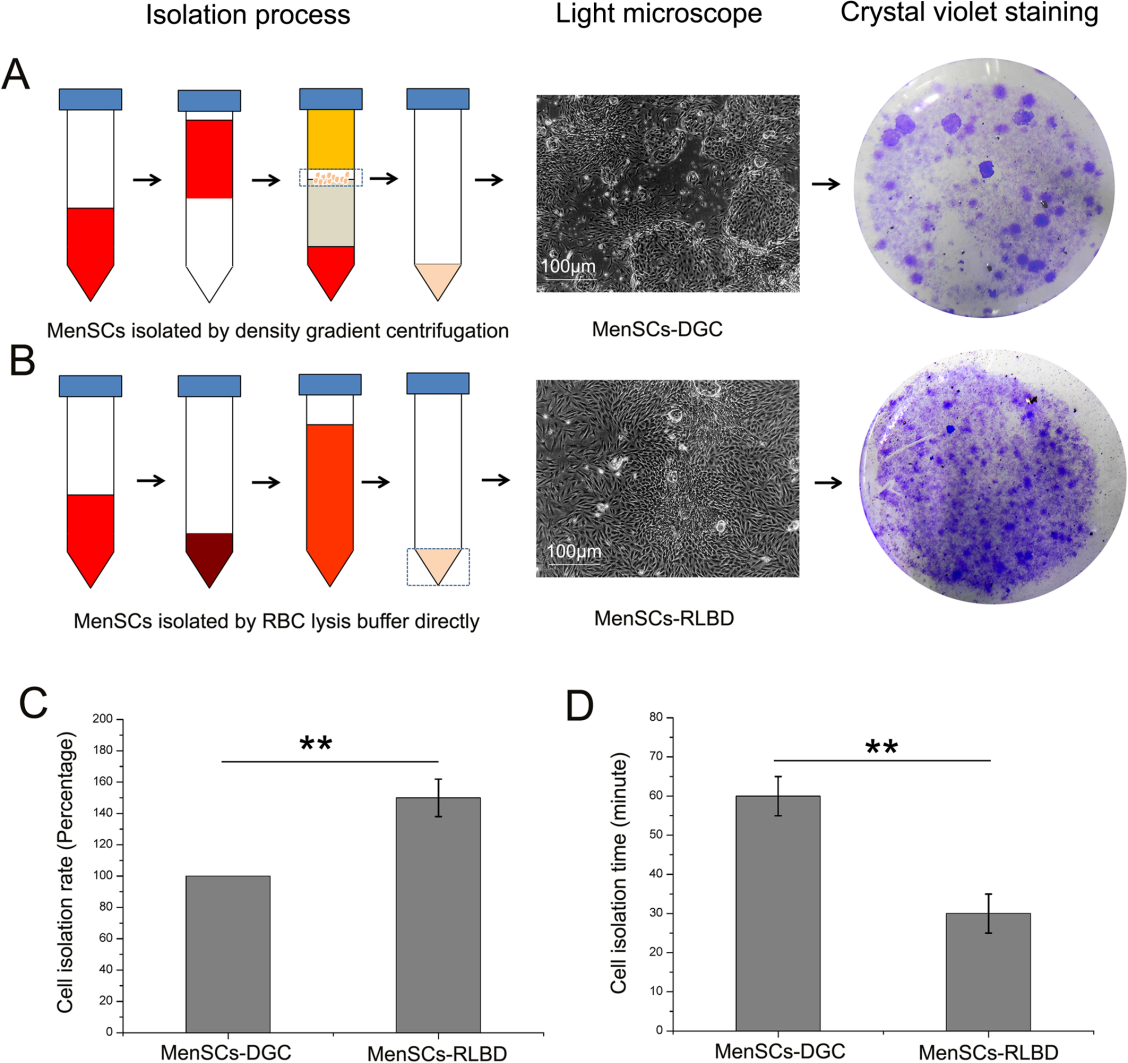
**MenSCs isolation efficiency analysis.** (A,B) The isolation procedures and morphology of primary MenSCs isolated by conventional DGC and RBC lysis buffer directly. After 5 days of culture, the morphology of MenSCs-DGC and MenSCs-RLBD clones was imaged under an inverted microscope and macroscopically observed after Crystal Violet staining. (C) After 5 days of culture, MenSCs-DGC (*n*=5) and MenSCs-RLBD (*n*=5) seeded in six-well plates were digested, and the number of cells was calculated by a cell counter. The cell isolation rate of MenSCs-DGC was taken as 100%, and the cell isolation rate of MenSCs- RLBD was calculated by the following formula: the quantity of MenSCs-RLBD/the quantity of MenSCs-DGC. (D) The time needed for MenSCs-DGC and MenSCs-RLBD isolation.

The above results have confirmed that there was a considerable amount of primary MenSCs remaining in the sedimentation after conventional DGC. To optimize the production of MenSCs, the isolation efficiency of both the DGC and RLBD methods was compared. As shown in Fig. 4C,D, the quantity of primary MenSCs isolated by RLBD was significantly increased (*P*<0.01) in comparison with that isolated by DGC from equal volumes of menstrual blood. Additionally, the isolation time for RLBD was significantly reduced (*P*<0.01) at half that of conventional DGC.

## DISCUSSION

MenSCs have become an attractive possibility for cell therapy in comparison to other stem cell types because of their abundance, high proliferative capacity, ease of periodic and non-invasive extraction and isolation, the absence of ethical limitations and the potential for autologous transplantation (Gargett et al., 2015; Ulrich et al., 2013). Due to these advantages, the use of MenSCs for cell therapy in various diseases is being extensively researched, and preclinical studies have shown beneficial effects in animal models of Duchenne muscular dystrophy (DMD), limb ischemia, myocardial infarction, stroke, sepsis, colitis, infertility and liver/lung injury after MenSCs transplantation (Gargett et al., 2015; Verdi et al., 2014; Khoury et al., 2014). Furthermore, clinical trials have also confirmed the improvement in the treatment for DMD patients, heart failure and severe Asherman's syndrome; additionally, as expected, no serious side effects related to MenSCs transplantation were observed in any patients during the one-year follow up after trials (Ichim et al., 2010; Tan et al., 2016).

It is now generally accepted that the prevailing therapeutic mechanism of ASC transplantation is primarily paracrine mechanism, which produces trophic factors and improves disease symptoms through angiogenesis, anti-apoptosis and immunoregulation. Recently, it has been demonstrated that MenSCs could ameliorate liver fibrosis by targeting hepatic stellate cells via paracrine bioactive factors, and the exosomes derived from MenSCs not only were able to alleviate fulminant hepatic failure but also showed beneficial effects in promoting neurite outgrowth (Chen et al., 2017; Lopez-Verrilli et al., 2016). Furthermore, research indicates that MenSCs may have superior paracrine properties compared with other ASC types, as evidenced by the fact that MenSC-derived improvement of cardiac function was principally, and perhaps exclusively, dependent on paracrine effects (Jiang et al., 2013). Our previous research further supports the superior paracrine activity of MenSCs, which showed high levels of angiogenic factors (VEGF, HGF, ANG and MMP-1) and cytokines (IL-6 and IL-8) secretions (Liu et al., 2018a).

However, one major impediment for ASC-based therapies is a high cell-death rate and poor cell engraftment at sites of damaged tissues, and it is reported that regardless of the delivery route (systemically or locally), only 1−5% of delivered ASCs reside in the target site for regeneration (Kurtz, 2008; Liu et al., 2018b). Consequently, the beneficial effects of ASC transplantation are likely to vanish as the stem cells disappear *in vivo* within a short time. An alternative strategy to overcome this challenge and maintain the therapeutic effects of ASCs is periodic transplantation, but this strategy requires sufficient amounts of the desired ASCs with a low number of subculture passages. Although abundance is one significant advantage of MenSCs, the volume of menstrual blood samples collected varies between different individuals (Salamonsen et al., 1999; Jarrell, 2018). Therefore, the optimization of the isolation of MenSCs can maximize the value of each sample and provide a substantial number of high quality MenSCs (with a low number of subculture passages) for clinical application, especially for patients whose menses have been seriously disturbed by the clinical treatment.

Based on experience and experimental results, we have demonstrated that primary MenSCs mainly reside in the deciduous endometrium. Thus, in this study, we first examined whether there were primary MenSCs remaining in the sedimentation (which contains the deciduous endometrium intertwined with menstrual blood clots) after DGC. Our results confirm the speculation that MenSCs can be harvested from this sedimentation using red blood cell lysis buffer, and the amount of harvested cells is considerable. As with MenSCs-DGC, the MenSCs-RLB possess typical morphology and mesenchymal stem cell surface markers, such as CD29, CD44, CD73, CD90 and CD105, but failed to express either hematopoietic cell markers CD34 and CD45 or HLA-DR. Moreover, embryonic stem cell markers including OCT-4, SOX2 and SSEA-4 were positively expressed in MenSCs-DGC and MenSCs-RLB, suggesting a superior multipotential of MenSCs; additionally, subsequent adipogenic and osteogenic differentiation further confirmed the multipotential of MenSCs.

The above results demonstrate that there is a considerable amount of MenSCs remaining in the sedimentation after DGC that are usually discarded as waste. Therefore, to improve the production of MenSCs, we bypassed the conventional DGC method and isolated MenSCs from menstrual blood by directly lysing red blood cells. As expected, the yield of MenSCs-RLBD increased approximately 1.5-fold compared to the yield of MenSCs-DGC alone; additionally, the time needed for isolation was shortened to half that of the conventional DGC isolation.

Taken together, our obtained results confirm that a substantial amount of primary MenSCs still remain in the sedimentation after conventional DGC, and indicate that MenSC isolation by directly lysing red blood cells not only collects a substantial number of high quality MenSCs with a low number of subculture passages, but also is timesaving and economical, providing solid support for extensive clinical application.

## MATERIALS AND METHODS

### Menstrual blood sample collection

The present study was approved by the Ethics Committee of the Xinxiang Medical University, China, and experimental procedures for menstrual blood samples and MenSCs were carried out in accordance with the approved guidelines. All the volunteer donors provided consent for the use of their menstrual blood samples in scientific research. Generally, the menstrual blood samples were collected with a DivaCup during the first 3 days of menstruation from five healthy volunteer donors (30±4 years, *n*=5). Subsequently, menstrual blood samples were transferred into a 50 ml tube and were immediately mixed with equal volumes of PBS containing 0.25 mg/ml amphotericin B (Sigma), 1% penicillin-streptomycin solution (100×, Genview) and 2 mM EDTA. Then, the samples were delivered to the laboratory, and the MenSC isolation was finished within 48 h.

### MenSCs isolation

Once receiving the menstrual blood samples in the laboratory, the MenSC isolation was immediately performed using the conventional DGC method described elsewhere with minor modifications (Liu et al., 2018a). Briefly, as shown in Fig. 1A, 5 ml of the diluted menstrual blood samples were gently added to centrifuge tubes preloaded with equal volumes of lymphocyte separation fluid; after centrifugation, the karyocytes and deciduous endometrium suspended in the buffy coat were transferred to new tubes, washed in PBS twice and suspended in growth medium [high-glucose DMEM medium (HyClone) supplemented with 10% FBS (Gibco) and 1% penicillin-streptomycin solution], and then seeded into a six-well plates or 25 cm^2^ plastic cell culture flasks incubated at 37°C with 5% humidified CO_2_. The MenSCs isolated by this method were labeled MenSCs-DGC.

Simultaneously, after the suspended karyocytes and deciduous endometrium were transferred into new tubes, the supernatant was completely removed, and 10 ml of 1× red blood cell (RBC) lysis buffer (155 mM NH_4_Cl, 10 mM KHCO_3_ and 0.1 mM EDTA-Na2, Genview) was used to suspend the sedimentation for 3–5 min at room temperature (20–25°C). The RBC lysis process was repeated twice until the majority of the erythrocytes were osmotically lysed by the ammonium chloride treatment. After two washings in PBS, the sedimentation was suspended in growth medium and seeded into six-well plates or 25 cm^2^ plastic cell culture flasks incubated at 37°C with 5% humidified CO_2_. The MenSCs isolated by this method were labeled MenSCs-RLB. After 2 days of culture, non-adherent cells were washed away with PBS leaving behind adherent fibroblastic cells growing in clusters. The growth medium was replaced every 3 days.

After 5 days of culture, the morphology of the MenSC clones grown in the six-well plates was imaged under an inverted microscope and macroscopically observed after Crystal Violet staining. Thereafter, when the cells seeded in the 25 cm^2^ plastic cell culture flasks reached 80%–90% confluence (Passage 0, P0), the cells were detached by 0.25% trypsin/1 mM EDTA and were subcultured in new flasks with a ratio of 1:3.

In addition, to evaluate the MenSC isolation efficiency between the DGC and RLBD (in which the MenSCs were isolated by RBC lysis buffer directly, Fig. 4B) methods, samples of menstrual blood collected from the same donor were equally divided into two parts. One part was used for conventional DGC MenSC isolation as described above. The other part was centrifuged to collect sedimentation, and the RBC lysis buffer was used to lyse the erythrocytes in the sedimentation. After three treatments, the remaining sedimentation was washed twice in PBS and seeded into six-well plates. The MenSCs isolated by this method were labeled MenSCs-RLBD. After 5 days of growth, the cells seeded in the six-well plates were digested by trypsin, and their number was calculated by a cell counter (Countstar, China), the cell isolation rate of MenSCs-DGC (*n*=5) was taken as 100%, and the cell isolation rate of MenSCs-RLBD (*n*=5) was calculated by the quantity of MenSCs-RLBD/the quantity of MenSCs-DGC. Additionally, conventional crystal staining was performed to macroscopically observe the MenSCs isolated by DGC and RLBD.

### Immunofluorescence

After 5 days' culture, P0 MenSCs-DGC (*n*=3) and MenSCs-RLB (*n*=3) were fixed with 4% PFA for 20 min and permeabilized with 0.05% Triton X-100 for 10 min, and nonspecific binding sites were blocked using 5% goat serum for 30 min. Anti-Nanog (Cat#: ab109250, dilution ratio 1:200, Abcam), anti-SOX2 (Cat#: ab92494, dilution ratio 1:100, Abcam), and anti-SSEA4 (Cat#: ab16287, dilution ratio 1:250, Abcam) were separately added, and the cells were incubated at 4°C overnight. Subsequently, Alexa Fluor 488-conjugated goat anti-mouse (Cat#: A-11001, dilution ratio 1:1000, Life Technologies) or anti-rabbit (Cat#: A-11034, dilution ratio 1:1000, Life Technologies) secondary antibodies were incubated with the cells at 37°C for 90 min. Cell nuclei were stained with DAPI. Finally, the cells were observed and imaged under a fluorescence inverted microscope (Leica), and photograph parameters were kept consistent in all the images for the further analysis. Briefly, after the original image was transformed to monochrome image, the green fluorescence intensity (gray value) of cell area in whole image was analyzed by ImageJ software, and the fluorescence intensity (gray value) in MenSCs-DGC was set to a value of 1. The data can indirectly reflect the expression of targeted protein in isolated MenSCs.

### Proliferation assay

To assess the proliferative capacity of MenSCs-DGC (*n*=5) and MenSCs-RLB (*n*=5), the P3 cells were suspended in growth medium and seeded at a concentration of 2.5×10^3^ cells/well into 96-well plates respectively, and the growth medium alone was set as background. After culturing for 1, 3, 5, 7 and 9 days, proliferative response was determined by a conventional MTT assay as described elsewhere, and absorbance was measured at 570 nm (reference wavelength: 630 nm) using an ELISA reader SpectraMax^®^ i3 (Molecular Devices). The Proliferation Indexes (PI) are presented as fold-change relative to the absorbance of MenSCs cultured for 1 day. PI=(Abs value of MenSCs cultured for different days−Abs value of background)/(Abs value of MenSCs cultured for 1 day−Abs value of background), and the PI of MenSCs cultured for 1 day has a value of 1.

### Flow cytometry

P3 MenSCs-DGC (*n*=5) and MenSCs-RLB (*n*=5) were used for the immunophenotyping analysis. Mouse anti-human monoclonal antibodies: FITC-conjugated CD29 (Cat#: 11-0299-42, dilution ratio 1:20, eBioscience), CD44 (Cat#: 11-0441-82, dilution ratio 1:20, eBioscience), CD73 (Cat#: 11-0739-42, dilution ratio 1:20, eBioscience), CD90 (Cat#: 11-0909-42, dilution ratio 1:20, eBioscience), HLA-ABC (Cat#: 11-9983-42, dilution ratio 1:20, eBioscience), HLA-DR (Cat#: 11-9956-42, dilution ratio 1:20, eBioscience), CD34 (Cat#: 11-0349-42, dilution ratio 1:20, eBioscience), CD45 (Cat#: 11-9459-42, dilution ratio 1:20, eBioscience) and PE-conjugated CD105 (Cat#: 12-1057-42, dilution ratio 1:20, eBioscience) were used. As negative controls, isotype PE (Cat#: 12-4714-42, dilution ratio 1:20, eBioscience) and FITC-conjugated IgG (Cat#: 31505, dilution ratio 1:20, eBioscience) were used. The cell suspensions (1×10^6^ cells) were washed twice with PBS and incubated with the monoclonal antibodies at 4°C in the dark for 30 min. After washing with PBS, the samples were resuspended and analyzed using a Cytomics FC 500 MPL cytometer (Beckman Coulter).

### Adipogenic and osteogenic differentiation

Adipogenic and osteogenic differentiation were performed, and the cell types were identified as described elsewhere. Briefly, for differentiation assays, P3 MenSCs-DGC (*n*=3) and MenSCs-RLB (*n*=3) were suspended in growth medium, seeded at a density of 2×10^4^ cells/well in a six-well plate and grown to 50% confluence. Subsequently, the growth medium was changed to either adipogenic differentiation medium (growth medium+1 μmol/l dexamethasone+10 µg/ml recombinant human insulin+200 µM indomethacin+0.5 mM IBMX, for 14 days) or osteogenic differentiation medium (growth medium+0.1 μmol/l dexamethasone+0.05 mmol/l ascorbic acid+10 mM β-glycerophosphate, for 21 days), the induction medium was replaced every 3 days. Control cells were cultured in growth medium. At the end of the induction periods, the cells were washed and fixed. Adipogenic differentiation was confirmed by Oil Red O staining and osteogenic differentiation was confirmed by Alizarin Red staining.

### Statistical analysis

The results were presented as the mean±s.d., and Student's *t*-test was used to determine statistical significance. *P*<0.05 was considered to be statistically significant.
